# Women in tunicate research: Pioneers of the past and their present legacy

**DOI:** 10.1002/dvg.23578

**Published:** 2023-11-27

**Authors:** Marie L. Nydam, Mary Beth Saffo, Anna Di Gregorio

**Affiliations:** 1 Life Sciences Concentration, Soka University of America, Aliso Viejo, California, USA; 2 Okemos, Michigan, USA; 3 Department of Molecular Pathobiology, New York University College of Dentistry, New York, New York, USA

**Keywords:** ascidians, developmental biology, taxonomy, tunicates, women scientists

## Abstract

The search for female scientists who pioneered the research on tunicates is hindered by the tradition of reporting only the first initials of authors’ names on scientific publications using only the initials of their first names. While this practice has the theoretical merit of broadening the readership by preventing the possible bias that could be caused by the gender of the author(s) in some of the readers, it rendered the identification of female researchers active in, or before, the first half of the 20th century quite challenging. Sifting through several dozen electronic records, and with the help of references and/or quotes found online, we have stitched together the information that we were able to retrieve on the life of female scientists who authored some of the earliest publications on tunicates, and we have organized them in (approximate) chronological order. We have also compiled brief synopses of the findings of scientists active in the field of tunicate biology in more recent times, and organized them by subdiscipline.

## 1 | EARLY PUBLICATIONS

### 1.1 | Edith Sumner Byxbee, 1873–1931

Edith Sumner Byxbee was born in February 1873, in California, USA, to Robert George Byxbee and Maria L. Spring. She graduated from Oakland High School in May of 1891 (van de Hoek, 2008a). After her high school graduation, she enrolled at the University of California (U.C.) at Berkeley, and while there she was affiliated with the California Academy of Sciences in San Francisco (van de Hoek, 2008a). By 1897, Edith completed the requirements for a Bachelor of Science degree, and in 1899 she graduated with a Master’s of Science degree from U.C. Berkeley. It is quite likely that her emphasis was Botany (van de Hoek, 2008a). She also took field trips to the Sierra Nevada, between 1895 and 1897, during which she collected bryophytes, and to Mendocino County, where she collected vascular plants that are now either part of the Jepson Herbarium or the University of California Herbarium (van de Hoek, 2008a). In 1900, 3 years after completing her university degree, Edith published her Master’s thesis in the Proceedings of the California Academy of Sciences (Byxbee, 1900). The focus of the study was spindle formation during meiosis in pollen mother cells of a plant in the genus *Lavatera* (Byxbee, 1900).

In 1905, she published a report on pelagic tunicates with William Emerson Ritter, the American biologist who in the early 1900s, together with Ellen Browning Scripps, formed the Marine Biological Association of San Diego (renamed as the Scripps Institution for Biological Research in 1912, and as the Scripps Institution of Oceanography around 1923; Ritter & Byxbee, 1905). The report focuses on specimens collected in the tropical Pacific Ocean by the US Fish Commission ship “Albatross” in 1899–1900 (Ritter & Byxbee, 1905). Ritter, a world expert on ascidians, and Byxbee, named a new species, *Pyrosoma agassizi*, a tunicate of the class Thaliacea, after the famous engineer and ichthyologist Alexander Agassiz, who had invited them to describe and classify the “Albatross” samples (Ritter & Byxbee, 1905).

Around this time, Byxbee became acquainted with Charles Palmer Nott, a naturalist and scientist affiliated with the California Academy of Sciences, and in January 1904 her engagement to Mr. Nott was announced during the “Gamma Phi Beta” sorority meeting at a home in the San Francisco Bay region (van de Hoek, 2008a). She married him soon afterward, and after that there is no further evidence of her scientific work (van de Hoek, 2008a). She had four children: Robert, Alison, Margaret, and Mary. She died in January 1931 at the age of 57, and was buried in Oakland, California.

Portions of the biographical information presented here were obtained from the archive of the *San Francisco Call*, and ancestors.familysearch.org.

### 1.2 | Myrtle Elizabeth Johnson, 1881–1967

**FIGURE 1:**
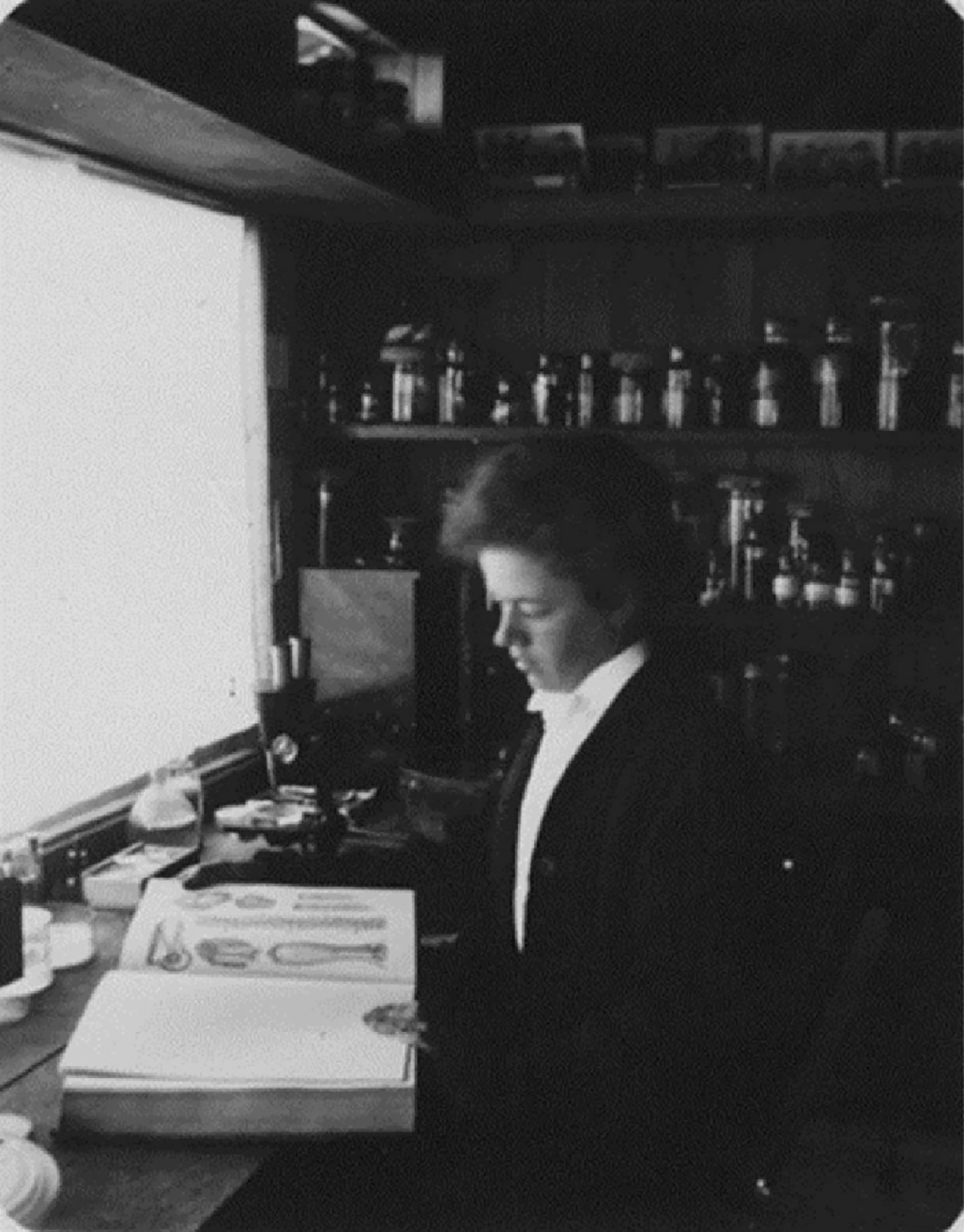
Myrtle Johnson, photographed at the laboratory of the Marine Biological Association of San Diego in 1905. https://library.ucsd.edu/dc/object/bb9455051v.

**FIGURE 2:**
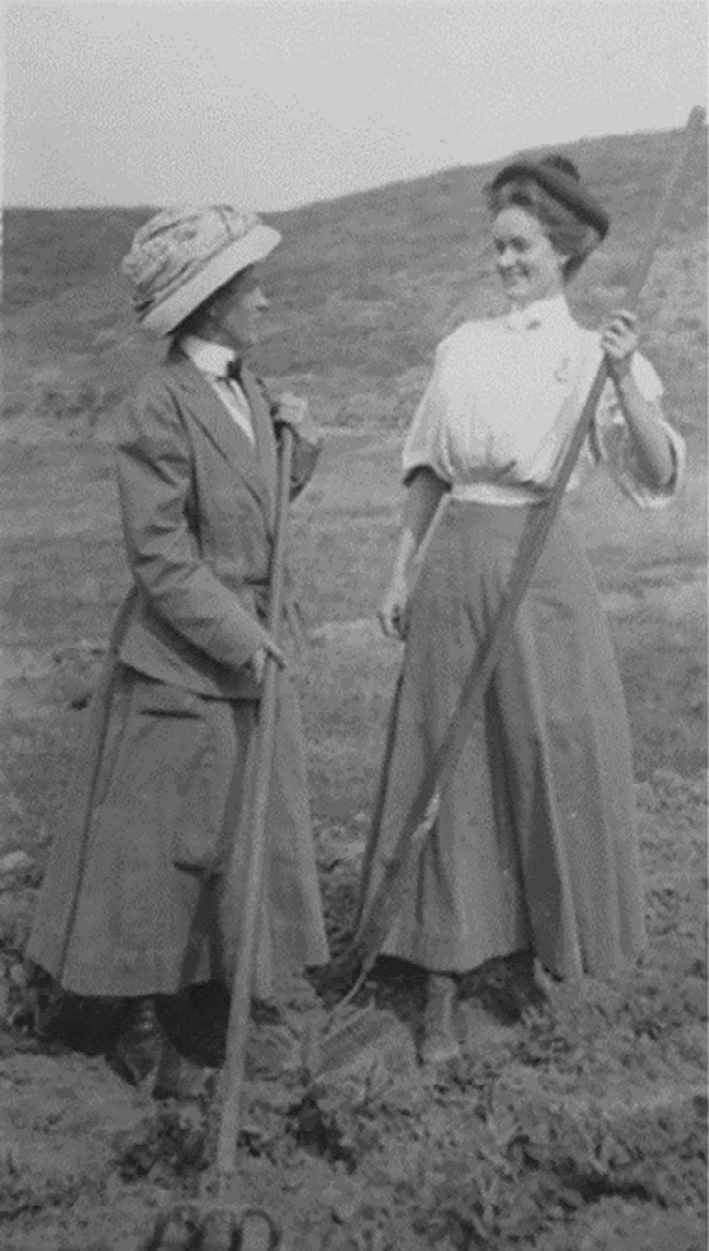
Edna Watson Bailey and Myrtle Elizabeth Johnson, circa 1910. https://library.ucsd.edu/dc/object/bb2867964b.

**FIGURE 3:**
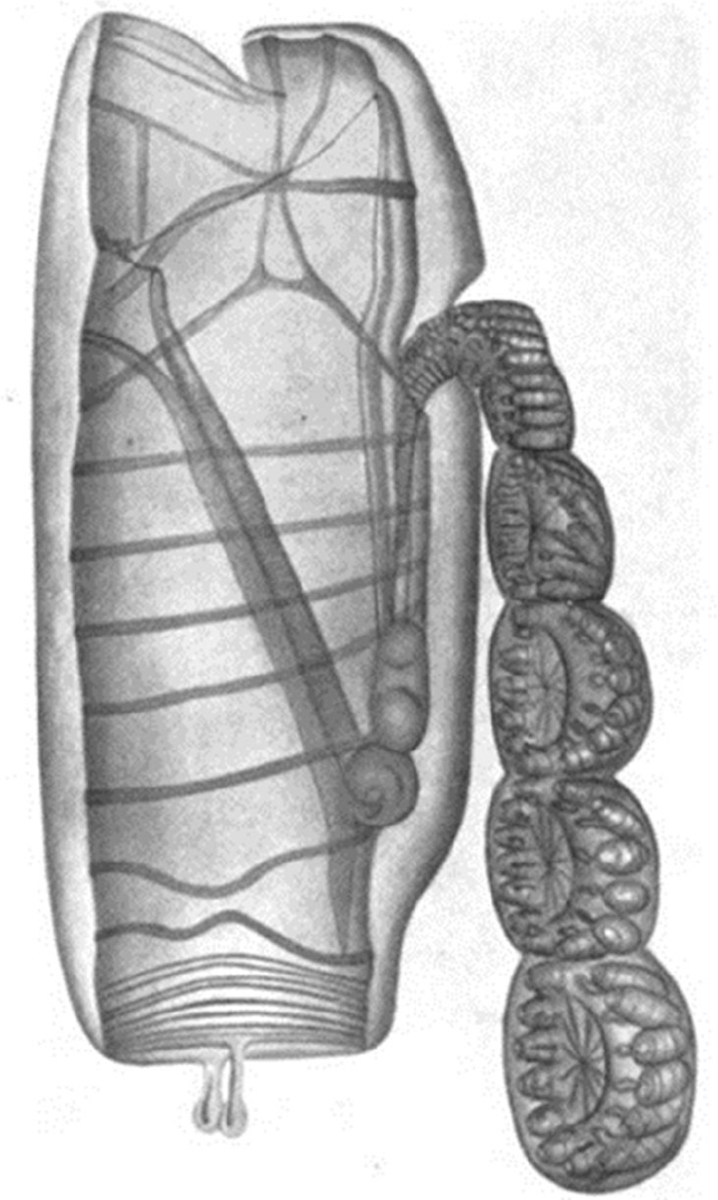
Plate 1 of Ritter & Johnson, 1912. *Cyclosalpa affinis* (Chamisso, 1819), solitary generation with a chain of five wheels.

Myrtle Elizabeth Johnson was born in 1881 and moved with her family to San Diego, California (USA) in 1887. In 1908, she earned a B.S. in Math and Zoology from U.C. Berkeley. She was photographed as an undergraduate around 1905 at the Marine Biological Association of San Diego (Figure 1), and with her friend and fellow educator Edna Watson Bailey in 1910 (Figure 2). For her Master’s degree at U.C. Berkeley, she focused on *Salpa fusiformis runcinata*, a pelagic tunicate (Johnson, 1909). She also published an article with William Emerson Ritter on salp development (Ritter & Johnson, 1912; Figure 3). In 1909, Ritter named an ascidian, *Halocynthia johnsoni*, in Myrtle’s honor (Ritter, 1909) for her excellent detailed scientific illustration of a particular ascidian that he was studying (van de Hoek, 2008b). Myrtle Johnson went on to complete a PhD at U.C. Berkeley in 1912, working with Harry Beal Torrey on pigment formation in amphibian larvae (Johnson, 1913). She was listed in 1912 as scientific staff at the newly founded Scripps Institution for Biological Research (Ritter, 1912). She was a long-time faculty member (1921–1946) and chair (1928–1940) of the Biology Department at San Diego State College (now San Diego State University) and the first female faculty member holding a PhD in the school’s history. She wrote the first dedicated guide to seashore life of the Pacific Coast in the 1920s, within a decade of completing her PhD (Johnson & Snook, 1927). The marine biologist Ed Ricketts, who published the successor to Johnson and Snook’s book, called their book “the *vade mecum* of marine biologists of the Pacific. Indispensable.” (Ricketts & Calvin, 1939).

Portions of the biographical information presented were obtained from Wikipedia.

### 1.3 | Ruth Agnes Forsyth, 1887 to unknown

Ruth Agnes Forsyth was born in 1887 and graduated from U.C. Berkeley in 1909 (van de Hoek, 2008c). From 1910 to 1915 she taught science and mathematics at the Nordhoff High School in Ojai, California (Bristol, 1939; Bristol, 1946).

After teaching high school, she enrolled in the graduate program in Zoology at U.C. Berkeley (van de Hoek, 2008c). The library of the University of California shows that she completed a Master’s thesis in 1916 (van de Hoek, 2008c). The research thesis involved an investigation of ascidians in the rocky intertidal and harbors of southern California (van de Hoek, 2008c). Her advisor was William Emerson Ritter (see above).

**FIGURE 4:**
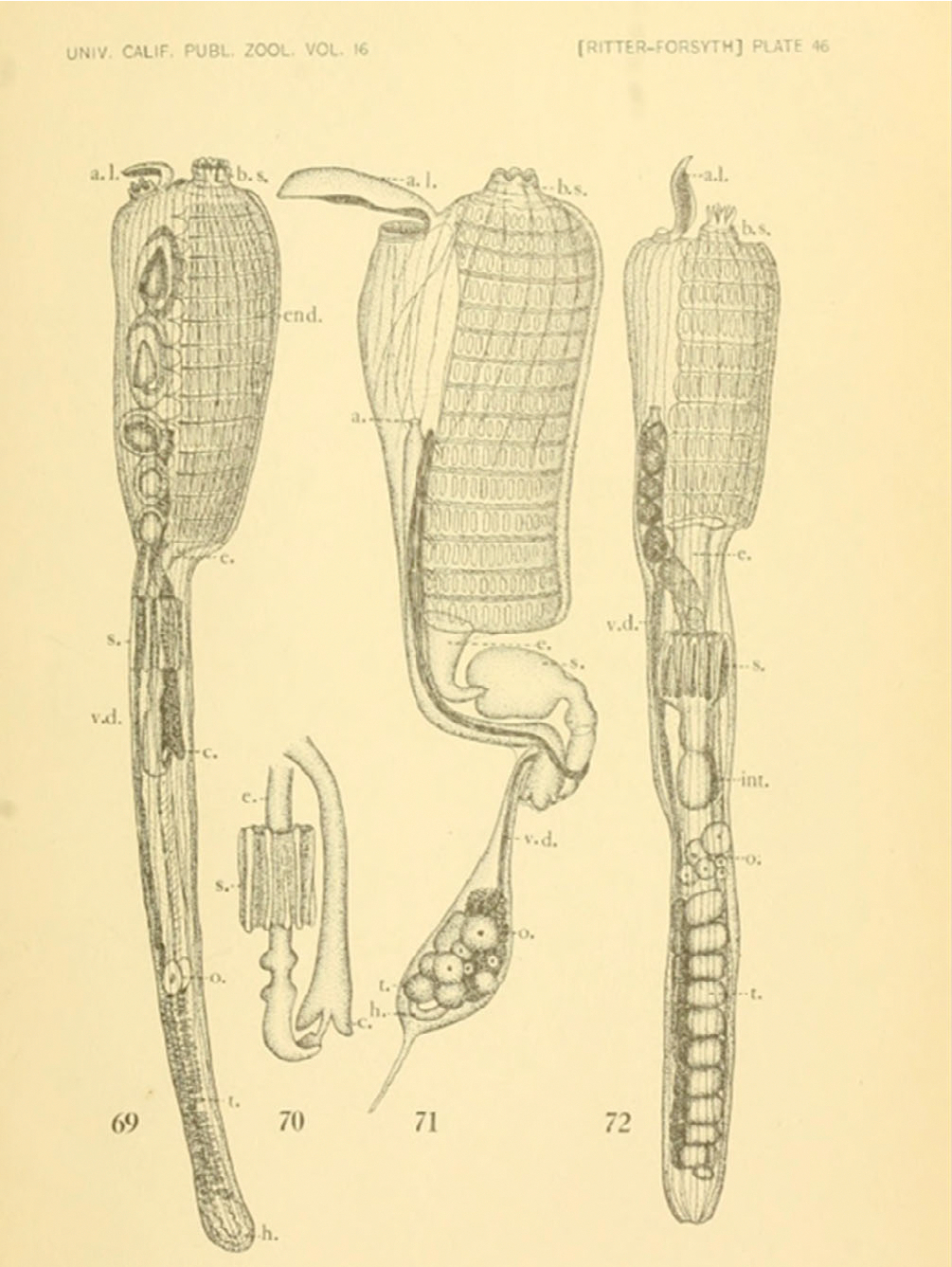
Plate 46 from Ritter & Forsyth, 1917. From the left: a zooid of *Aplidium solidum* (formerly *Amaroucium solidum*), the stomach of *Aplidium solidum* (formerly *Amaroucium solidum*), a zooid of *Polyclinum planum*, and a zooid of *Aplidium californicum* (formerly *Amaroucium californicum*).

A year after the completion of her Master’s thesis, she and Ritter coauthored a peer-reviewed scientific report on shallow-water ascidians of southern California that appeared in the *University of California Publications in Zoology* (Ritter & Forsyth, 1917). Ascidian populations south of Point Conception were understudied at the time, and this publication therefore filled a large gap in knowledge (van de Hoek, 2008c). The authors conducted extensive field and museum work to create a list of all the species found in the area (Ritter & Forsyth, 1917). For each species, they described the external characteristics, branchial, digestive and reproductive systems, habitat, and distribution (Ritter & Forsyth, 1917); in addition, this article provides a description of the morphological characteristics that define each genus. Fifteen new species were described in this exceptional work, including *Botrylloides diegensis* (Ritter & Forsyth, 1917), which is now found worldwide in temperate zones. In total, this publication contains 72 exceptionally detailed illustrations (Figure 4). This report was heavily quoted by Van Name in his 1945 monograph “The North and South American Ascidians,” and is still an invaluable taxonomic and ecological resource for ascidian researchers today. The distribution information provides a baseline from which to track the changes in ascidian species over the past 100 years. Many of the species that were easily found in the intertidal zones by Ritter and Forsyth are absent today (M. Nydam, personal communication), and the native species living on artificial structures have all been replaced by non-native species, except for *Ascidia ceratodes* (Lambert & Lambert, 2003).

### 1.4 | Gladys Amelia Anslow, 1892–1969

**FIGURE 5:**
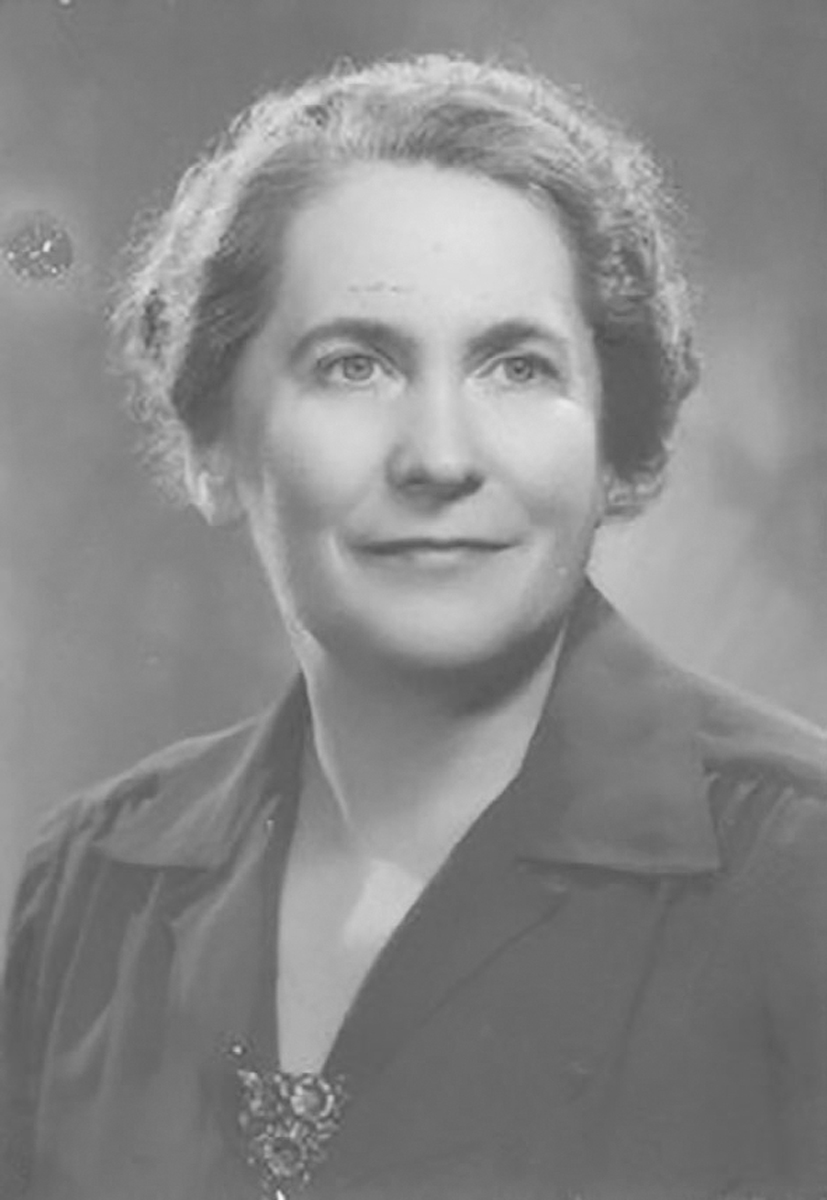
Gladys Anslow. Contributed by Ed Pomeroy (Anslow, n.d.).

Dr. Anslow (Figure 5), a prominent physicist, was born in Springfield, MA (USA) and matriculated to Smith College in 1909. At Smith, she was a member of the Mathematical Society and a Vice President of the Physics Club. She obtained her BA in 1914, and continued to work at Smith College, as a Physics demonstrator and assistant (1914–1917). She also obtained her Master’s degree at Smith College, working with Prof. Janet Howell Clark, physiologist and biophysicist (The Smith Alumnae Quarterly Alumnae Association of Smith College, 1917). Her thesis, titled “Spectroscopic Evidence for the Electron Theory of Matter”, focused on the emissions spectra of radium. She obtained her PhD from Yale University in 1924, and published this work in *Science* in the same year (Anslow, 1924). After obtaining her PhD, Dr. Anslow returned to Smith College as an associate professor and became a full professor by 1930. In 1940, she became the director of graduate studies at Smith College. She was the first woman to work with the cyclotron at U.C. Berkeley and one of the few women involved in the Manhattan project during World War II. Towards the end of World War II, she was the chief of the office that decided how and when classified information was communicated to researchers (Howes & Herzenberg, 1999). She was given the President’s Certificate of Merit for her assistance to the U.S. government during World War II. Later in her career, she published with Otto Glaser experiments that were carried out at Woods Hole Marine Biological Laboratory on the effects of copper on ascidian metamorphosis (Glaser & Anslow, 1949). This work was important because copper paint is very commonly used to prevent biofouling but has negative environmental and ecological consequences (Cima & Varello, 2023). From a developmental biological perspective, this article was influential because it introduced the concept of “promoters of metamorphosis,” which was cited in subsequent publications (e.g., Berking & Herrmann, 1990).

Portions of the biographical information presented were obtained from Smith College Special Collections (Anslow, n.d.), Wikipedia, and references therein.

### 1.5 | Emma Catherine Herdman, 1899–1953

**FIGURE 6:**
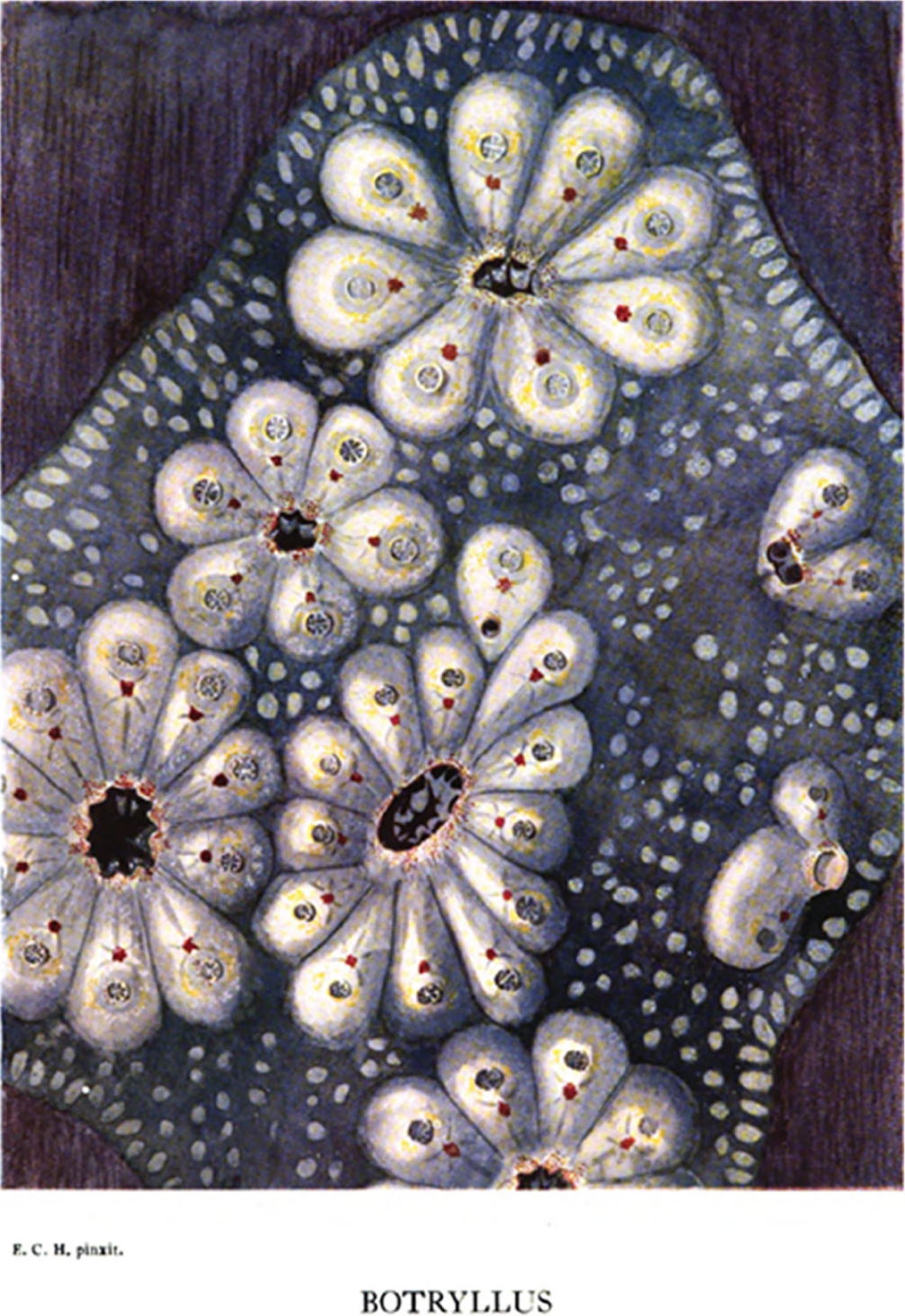
Emma Catherine Herdman’s painting of *Botryllus*. From Herdman (1924). https://books.google.com/books?id=dPhLAAAAMAAJ&printsec=frontcover&source=gbs_ge_summary_r&cad=0#v=onepage&q&f=false.

Emma Catherine Herdman was the daughter of Sir William Abbott Herdman and Jane Brandreth Holt. Ms. Herdman’s father, William, was a renowned marine biologist who contributed greatly to ascidian taxonomy, and her mother studied physics at the University of Liverpool, graduating with first-class honors (equivalent to *summa* cum *laude*; Draper, 2023). Ms. Herdman obtained a Master’s degree and worked as a volunteer in the Department of Natural History (later renamed Department of Zoology) of the University of Liverpool, where her father was a professor (Peaker, 2021). Her father retired in 1919, but she continued to work there as a volunteer researcher (Peaker, 2021). She authored a book on *Botryllus*, published in 1924, with beautiful illustrations, including her handmade painting of *Botryllus* (Figure 6; note the inscription underneath the figure: “E.C.H. *pinxit*,” her initials followed by the Latin word for “painted”). In the preface of her book, she acknowledges her father for his “constant helpful criticism and advice throughout my work” (Herdman, 1924).

Of note, Ms. Herdman worked closely with Ruth Culshaw Bamber, MSc 1914, lecturer in Zoology at the University of Liverpool, who became later known as “Mrs. Bisbee” and had worked in the past on marine organisms (Peaker, 2021). Along with Ruth Bamber, Ms. Herdman published several papers from 1922 to 1933 on the genetics of traits in cats, such as the Manx tail and the tortoiseshell color pattern (Bamber & Herdman, 1927a, 1927b, 1928, 1931a, 1931b, 1932). From 1930 until her untimely death from an autoimmune disease in 1953, she was an honorary lecturer in the Department of Zoology (Peaker, 2021). Emma Catherine Herdman never married, but she adopted three children (two boys and a girl) who took her family name (Peaker, 2021). Two genera of ascidians are named after her father and her: *Euherdmania* and *Herdmania* (Ritter, 1904).

Portions of the biographical information presented were obtained from geni.com.

### 1.6 | Sister Florence Marie Scott, 1902–1965

**FIGURE 7:**
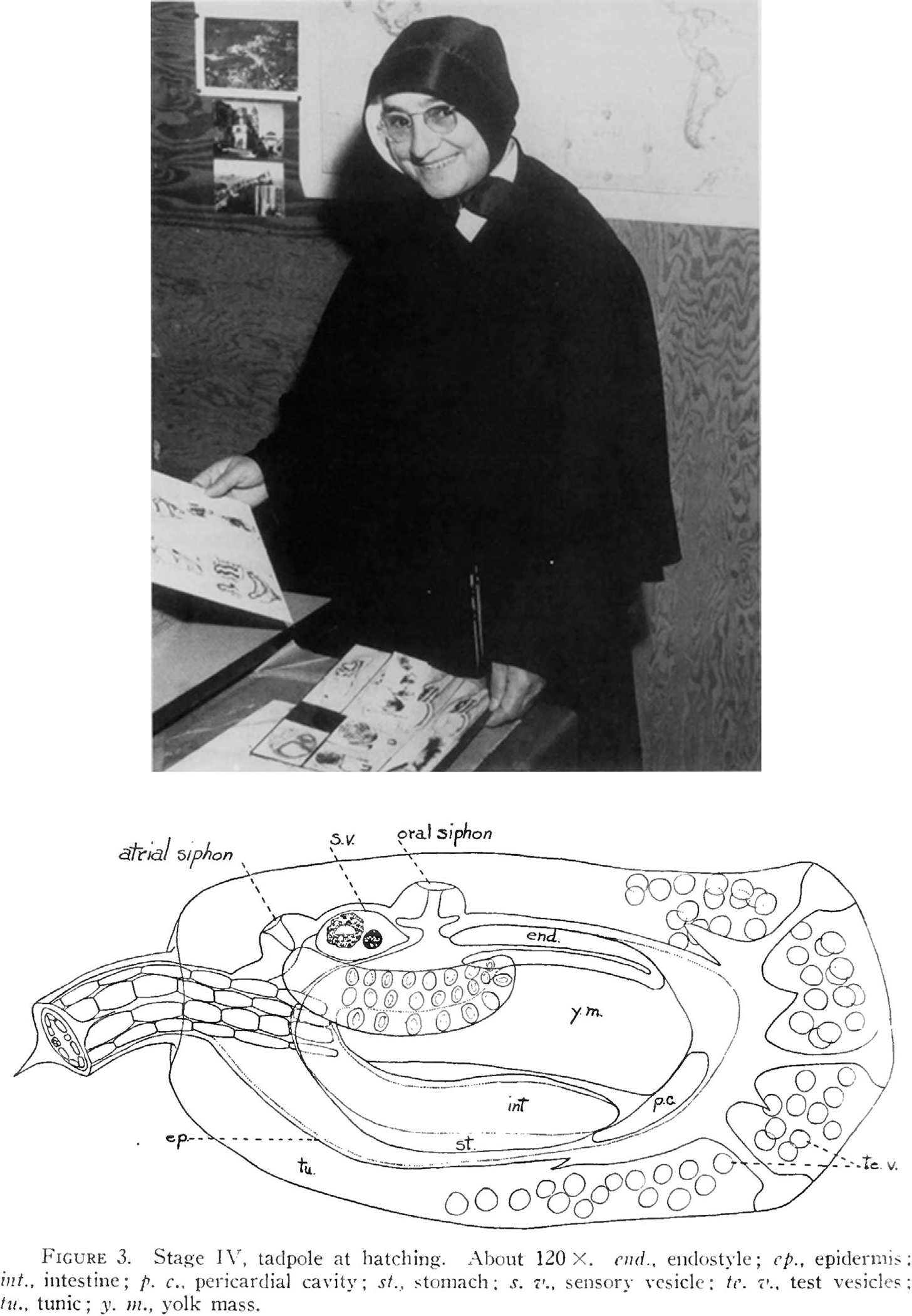
Sister Florence Marie Scott at Seton Hill University. Top: Sister Florence Marie Scott posing with two sets of her original drawings of tunicate development. Source: https://shualumni.setonhill.edu/ Bottom: Drawing of the *Amaroecium constellatum* tadpole at the time of hatching. From Scott (1946).

Florence Marie Scott was born in Johnstown, PA (USA) in 1902. After becoming a member of the Roman Catholic religious order of the Sisters of Charity, she graduated in 1926 from Seton Hill University, a Catholic liberal arts university founded by the Sisters of Charity and chartered in 1918. She then moved to Columbia University, where she received a Master’s degree in 1927 and a PhD in 1935. While at Columbia, she was taught by biologists who were also involved in teaching and research at the Marine Biological Laboratory (MBL) in Woods Hole, MA (USA). In 1929 she went back to Seton Hill and began her career as a professor in the Department of Biology; during the same year, she enrolled in the MBL Embryology Course and continued to spend her summers in Woods Hole for over 30 years after that. In Woods Hole, she studied tunicate biology, and in particular the development of *Botryllus schlosseri* and *Amaroecium constellatum* (Scott, 1934, 1945, 1946, 1952). Dr. Scott became very popular at the MBL and in the surrounding town, eventually becoming a member of the MBL corporation and, later on, a trustee (http://comm.archive.mbl.edu/publications/women_scott.html). She also rose through the ranks at Seton Hill, where she became full professor and chair of the Department of Biology (Figure 7). Later during her career, her research on tunicates focused on studies of regeneration and tissue differentiation (Scott, 1957).

### 1.7 | Nel E. Krijgsman: Active 1950–1959

Born Nel E. de Graaf, in Utrecht, Holland, she married B. J. Krijgsman and published with him as either Nel or Nelly E. Krijgsman while they both worked in the Department of Zoology at the University of Cape Town. They published in *Nature* a manuscript on the heart of arthropods (Krijgsman & Krijgsman, 1950) and in 1959, they published their investigation on the heart of *Ciona* (Krijgsman & Krijgsman, 1959).

In particular, they investigated whether the beating of the *Ciona* heart is due solely to intrinsic pacemakers or also to extracardiac regulation transmitted by nerve fibers entering the heart from outside. “Freshly collected specimens of *Ciona intestinalis* of 8–10 cm length when extended were used for the experiments. After slitting open the test and muscular body wall in the heart region, the heart was gently pulled out and turned over without separating it from the circulatory system” (Krijgsman & Krijgsman, 1959).

**FIGURE 8:**
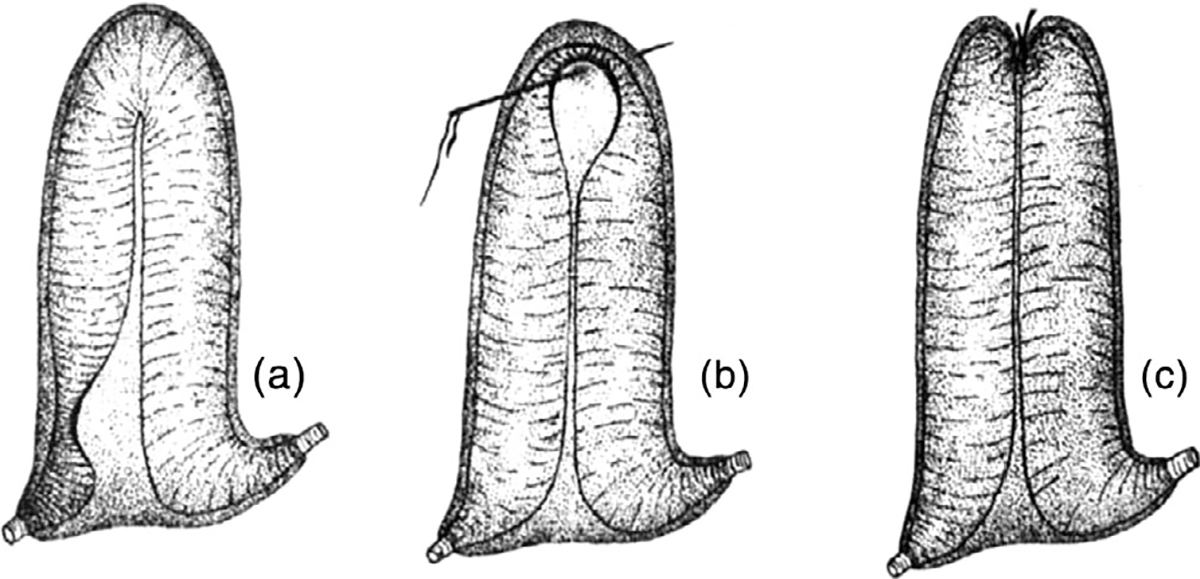
Ligature of the *Ciona* heart for studies of its pacemakers. From (Krijgsman & Krijgsman, 1959). See also Evans Anderson and Christiaen (2016).

They decided to work with isolated pacemakers, because “When using the whole heart in which the pacemakers influence each other (reversal of beat), one never knows how active one pacemaker would have been if it were not suppressed during the dominance of the other.” To accomplish that, “A fine needle was then inserted through the pericardium […] The needle, carrying very thin nylon thread, was then gently pulled through completely, and a ligature made (Figure 8). With two manually operated signal keys, one for the visceral, and one for the hypobranchial pacemaker, the frequency of each pacemaker could be recorded separately.” This study also tested the action of acetylcholine (ACh) and concluded that “the effect of ACh on the *Ciona* heart is by no means impressive,” which argued against the hypothesis of extracardial colinergic innervation. Of note, Dr. Lindsay Waldrop’s lab (Biological Fluid Dynamics at Chapman University) currently uses the valveless tunicate heart of *Ciona* to study the role of additional cardiovascular structures (such as the pericardium, the outer layer that surrounds the muscular tube), the pumping mechanisms (peristalsis, dynamic suction pumping), and the evolution of these structures (https://waldroplab.com).

### 1.8 | Winona Bethune Trason, 1925–1991

**FIGURE 9:**
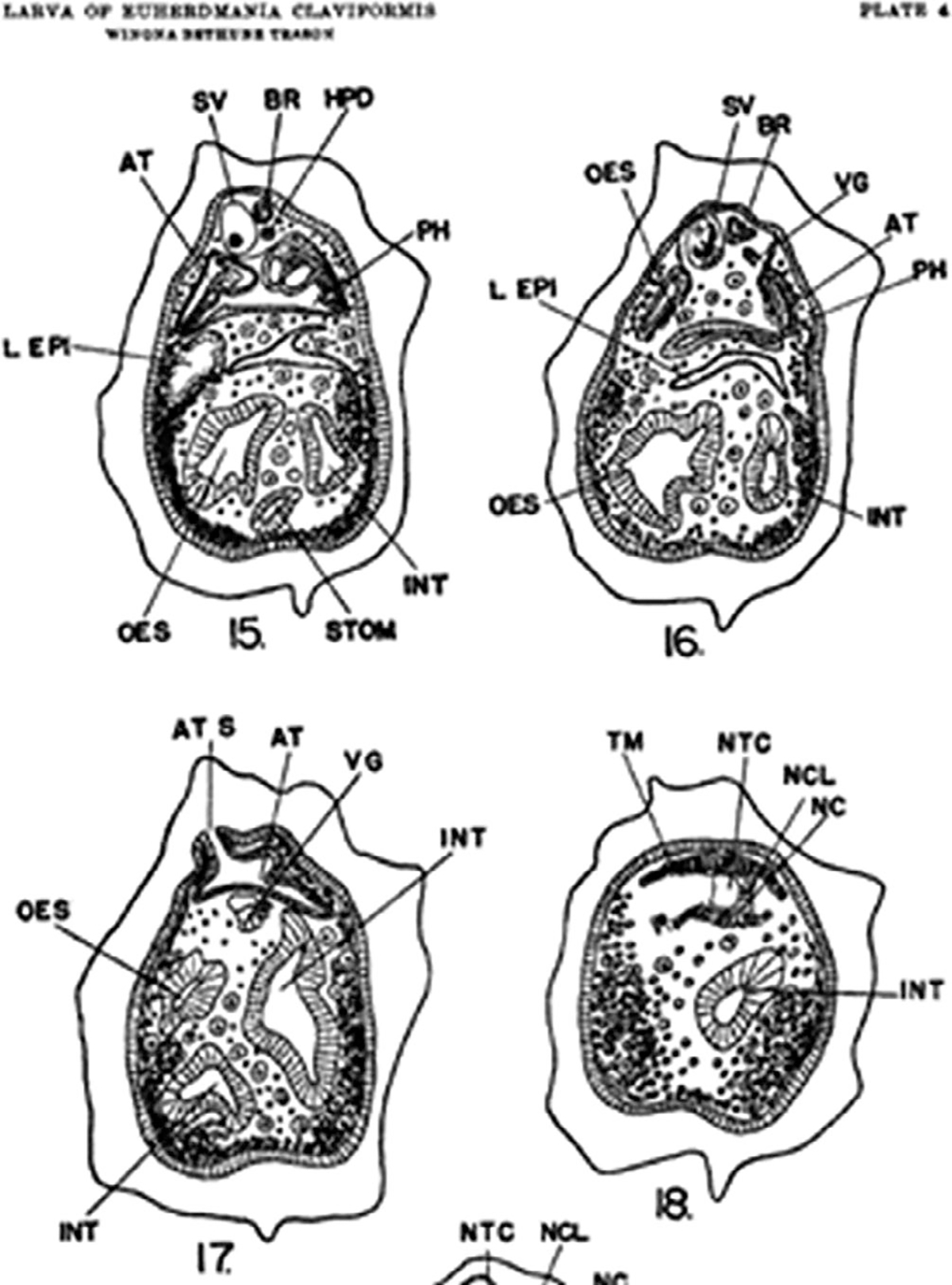
Transverse sections of a mature free swimming larva of *Euherdmania claviformis*. From Plate 4 of Trason (1957).

Winona Bethune Trason started her career with a focus on the reproduction and development of a common intertidal colonial ascidian, *Euherdmania claviformis* (Bethune, 1955). She continued to work on the larval and juvenile stages of this species (Trason, 1957; Figure 9), and subsequently on the ecology of the colonial species *Pycnoclavella stanleyi* (Trason, 1959). She worked with the invertebrate biologist Dr. Ralph Smith at U.C. Berkeley for her PhD, which she earned in 1960 (Hermans, 1997). She published a survey of the ascidians of the Canadian Arctic Waters in 1964 (Trason, 1964). In 1968, she published a description of two new species from Central California, *Ritterella rubra* and *Distaplia smithi*, with another former graduate student of Dr. Smith, Donald Abbott (Abbott & Trason, 1968). Dr. Trason taught in the Biology Department at Monterey Peninsula College, where there are currently two scholarships in her name, the Dr. Winona Trason Biology Scholarship and the Dr. Winona Trason Scholarship Fund (MPC Foundation, n.d.; mpcfoundation.org).

She and her husband, Dennis, a high school teacher, had two children, Ann and John (Dennis Trason 1926–2018). Her daughter, Ann Trason, is one of the most accomplished female ultramarathon runners of all time and has followed in her mother’s footsteps in teaching science to undergraduates.

Portions of the biographical information presented here were obtained from Wikipedia and references therein.

## 2 | CELL BIOLOGY, DEVELOPMENT, AND PHYSIOLOGY

### 2.1 | Helga Henriette Lindel Zwillenberg, 1930–2013

**FIGURE 10:**
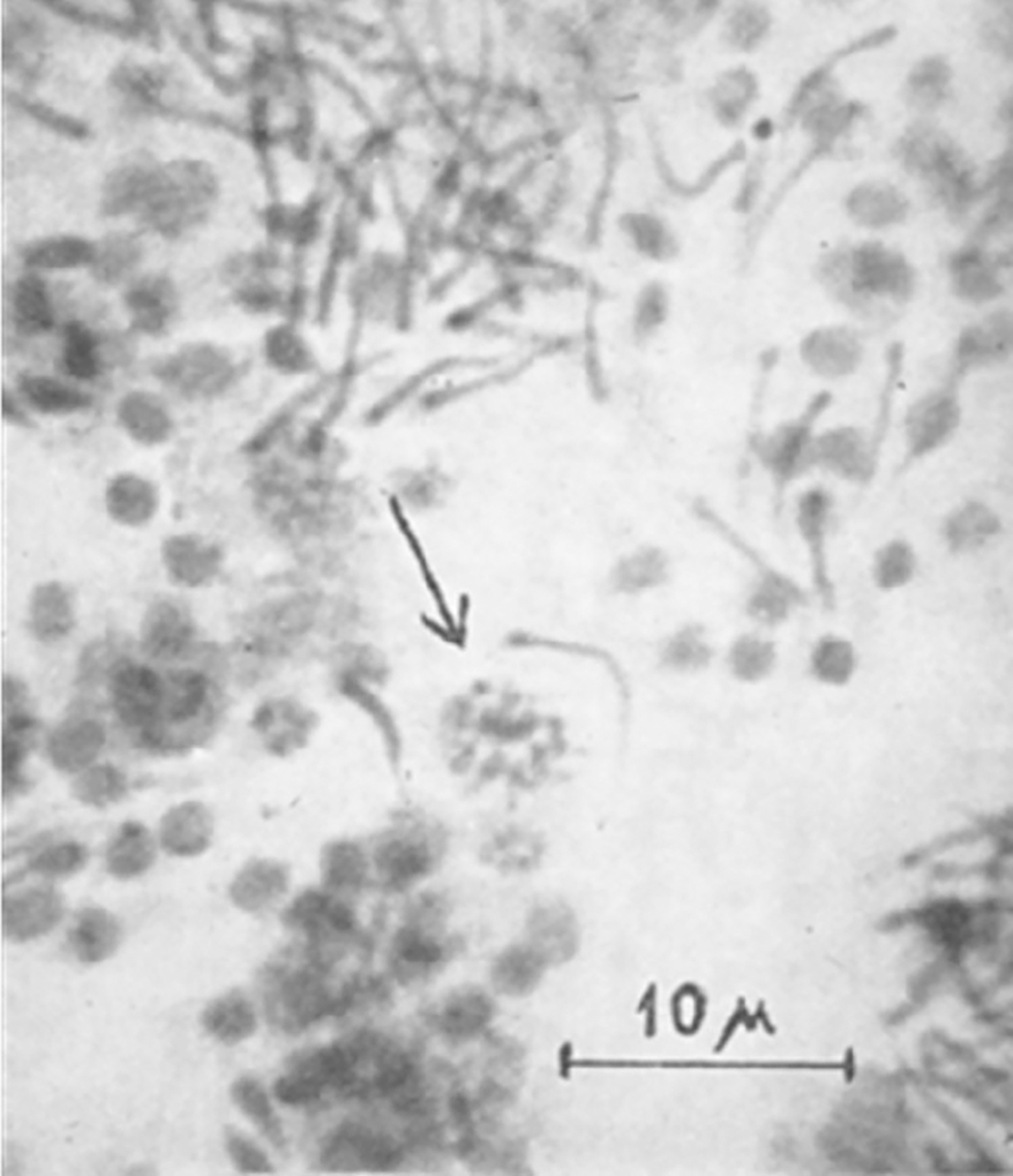
Meiosis in the ascidian *Sidnyum elegans*. From Zwillenberg and Zwillenberg (1954).

Helga Zwillenberg lived in Berlin with her parents Hugo, a German-Jewish diplomat and entrepreneur, and Elise Regina Tietz, the daughter of a department store entrepreneur. In addition to writing a book on the structure and function of the spleen (Zwillenberg, 1964) Helga carried out original research with her brother, Lutz Oscar, with whom she published at least 10 manuscripts, including a *Nature* paper reporting a method for squash preparations of tunicate chromosomes (Zwillenberg & Zwillenberg, 1954; Figure 10). A notable quote from the Zwillenbergs’ manuscript: “So far as is known to us this is the first instance in which chromosomes have been observed in tunicates. Although the tunicates are far from ideal for cytogenetical work, on account of the small size of the chromosomes, such cytogenetical work does not seem impossible.” Successful preparations of ascidian chromosomes were obtained several years later by Ivana Lazzaretto-Colombera and her collaborators (Institute of Animal Biology, University of Padova, Italy) for *C*. *intestinalis* and *Diazona violacea* (e.g., Colombera et al., 1978).

### 2.2 | Estees Potter Levine: Published 1960–1963

Dr. Levine earned her PhD from Stanford University after studying a broad range of ecological and developmental characteristics in the colonial ascidian *Eudistoma ritteri*, Van Name, 1945 in the laboratory of Donald P. Abbott, a professor whose main focus was the study of ascidians (seaside.stanford.edu/dpabbott; Levine, 1960, 1962). Soon after graduating from Stanford, she took a job as a professor of Anatomy and Physiology in the Health and Hygiene Department at San José State College in San José, California (USA). She was an assistant professor in 1960, and an associate professor by 1963 (The Daily Spartan). She further explored the metal accumulation of *E*. *ritteri* in a publication in *Science* (Levine, 1961). This work was highlighted in her College’s journal, the Daily Spartan, on October 24, 1963. Even though the first page of this newspaper is dominated by the photos of the five women selected as homecoming finalists, most of the second page of the daily newspaper is occupied by a laudatory article dedicated to the “red-haired physiologist” and her research on “a lowly sea animal that absorbs rare metals.” The article also mentions that Dr. Levine had been invited to present her findings at the American Association for the Advancement of Science (AAAS) annual meeting convention that was to be held in Cleveland in December of that year. Dr. Levine discovered, through spectrographic analysis, that in addition to being able to accumulate small amounts of vanadium, a peculiar feature that had been previously reported by other researchers, ascidians absorb chromium and titanium from the sea water. She connected the uptake of rare metals to the synthesis of hormones, which was another focus of her research, and formulated the hypothesis that “the hormones and metals work together in the replication of new tissues.” The article also summarizes her studies on ascidians found in European waters, which led her to discover that “a European species of sea squirt that lives in the English Channel and the Bay of Naples absorbed the rare metals and reacted to the hormone experiments.” This species is *D*. *violacea*, which in the abstract of her AAAS presentation is described as “a phlebobranch ascidian which does not occur in American waters” and is similar to the aplousobranch *Eudistoma*, which is found on the California coast (Levine, 1963). The gonads of both *Diazona* and *Eudistoma* differentiated in response to the treatment with mammalian gonadotropins (Levine, 1963).

### 2.3 | Clare M. Peddie: Active 1992–2002

The ascidian “blood” is the subject of a wealth of past and present studies, which have elucidated different types of circulating cells and documented their properties. A thorough study of the blood cell types in *C*. *intestinalis* (currently *Ciona robusta*) can be found in the PhD thesis written by Dr. Clare M. Peddie, currently a professor of Biology at the University of St. Andrews, in Scotland (UK). This very interesting thesis, titled “Lymphocyte-like functions in the solitary ascidian ‘*C*. *intestinalis*’” is publicly available online, and contains original light and transmission electron photomicrographs of different blood cells found in this ascidian, as well as a series of experiments aimed at monitoring the effects of treating these cells with different chemical compounds. Dr. Peddie’s thesis also reports the results of experiments focused on the cytotoxic activity of the *Ciona* hemocytes against mammalian target cells, which is compared with the cytotoxic activity of mammalian NK cells (Peddie & Smith, 1993, 1994). The mechanisms of phagocytosis and encapsulation were also investigated, in vitro, using either mixed populations or fractions of blood cells (Smith & Peddie, 1992). Clare Peddie and her collaborators also investigated phenoloxidase (PO) activity in the blood of *Ciona*, linked it to a specific type of blood cells, the “morula” cells, and studied its presence in eight additional ascidian species, reaching the conclusion that the PO system is widely used across different genera of ascidians (Jackson et al., 1993).

### 2.4 | The ascidian nervous system before and after metamorphosis

Before metamorphosis, ascidian larvae recapitulate the prototypical chordate body plan. Their tail contains a distinguishable notochord, and dorsal to it, an essential central nervous system (CNS) composed of a limited number of neurons and their accessory “ependymal” (non-nervous) cells. The laboratory of Prof. Ian Meinertzhagen at Dalhousie University, in Halifax, Nova Scotia (Canada) has greatly contributed to our understanding of the organization and function of the larval nervous system at the individual cell level, in particular through the work of four of its alumnae.

Dianne Nicol studied the lineage of the developing *Ciona* neural plate using scanning electron photomicrographs of embryos fixed at 12-min intervals from gastrulation through neurulation (Nicol & Meinertzhagen, 1988a; Nicol & Meinertzhagen, 1988b). Later on, by preparing longitudinal semithin sections of larvae and counting the number of nuclei present on each section, she determined that the average number of cells in the nervous system of the *Ciona* larva is ~335, and that 65 or 66 of these cells reside in the larva’s tail (Nicol & Meinertzhagen, 1991). She also concluded that ~68% of the cells of the larval nervous system are non-nervous, while the remainder are neurons. In addition, she counted and classified the cells that compose the larval sensory organs (Nicol & Meinertzhagen, 1991).

Alison Cole used confocal image stacks of whole-mount *Ciona* embryos from neurula to hatched larva to follow the mitotic activity and trace the lineage of the neural precursors that form the caudal derivative of the neural tube, the so-called nerve cord, and the “visceral ganglion,” which is the center that coordinates the swimming movements and is enriched in motor neurons (Cole & Meinertzhagen, 2004; see also Stolfi & Levine, 2011).

Janice H. Imai electroporated *Ciona* zygotes with a construct containing the promoter region of the neuronal marker *synaptotagmin* fused to the GFP reporter, to specifically label and follow different subtypes of neurons of the visceral ganglion and nerve cord. In particular, she identified at least four different subtypes of motor neurons, and for one of these subtypes, which she named contrapelo cells, she provided a first description of their projections (Imai & Meinertzhagen, 2007a). Using the same approach, she also identified different subpopulations of peripheral neurons, some of which are found in the papillae of the rostrally located adhesive organ while the remaining ones are interspersed throughout the epidermis (Imai & Meinertzhagen, 2007b).

**FIGURE 11:**
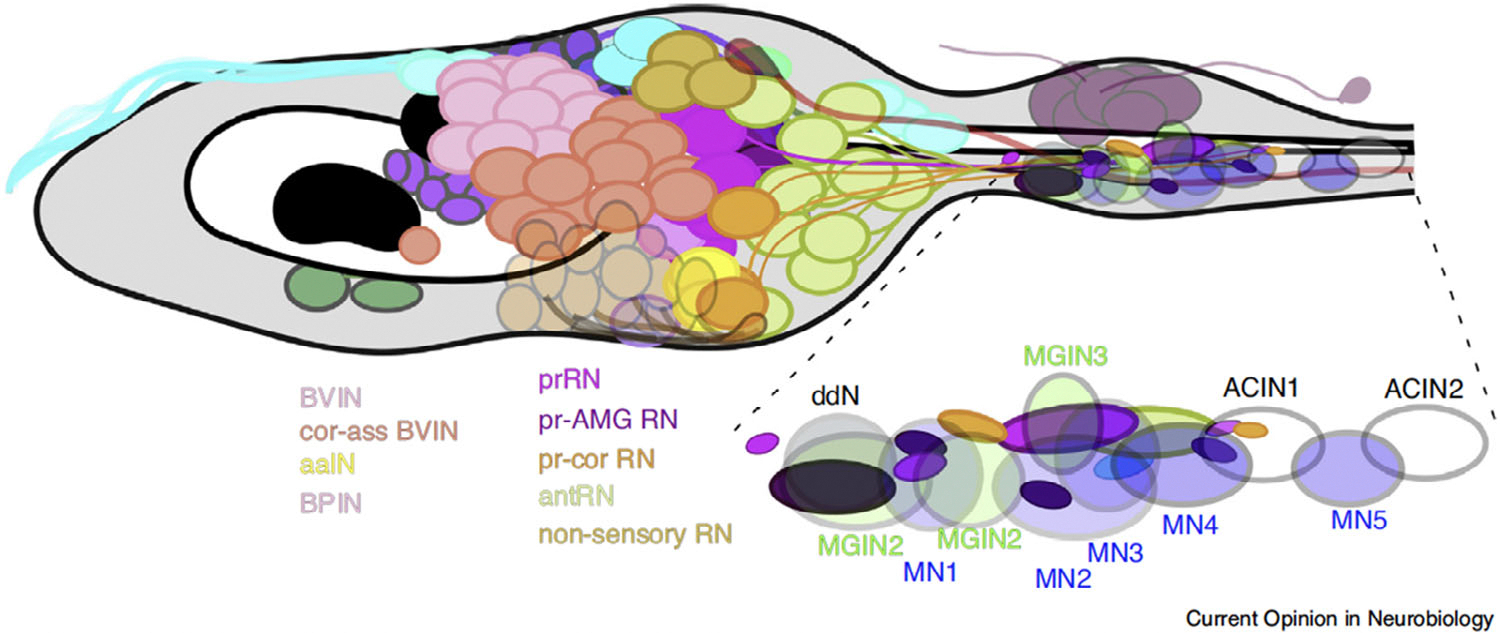
Map of the larval nervous system of *Ciona*, obtained from the studies carried out by alumnae of the Meinertzhagen lab. Drawing of the brain vesicle of a *Ciona* larva and part of its tail. The region of the motor ganglion is magnified on the lower right. Neurons are symbolized by ovals, color-coded on the base of their respective subtypes. Anterior is on the left. aaIN, anaxonal arborizing intrinsic interneuron; ACIN, ascending contralateral inhibitory neuron; antRN, antenna relay neuron; BPIN, bipolar brain vesicle interneuron; BVIN, brain vesicle interneuron; cor-ass BVIN, coronet-associated brain vesicle interneuron; ddN, descending decussating neuron; MGIN, motor ganglion descending interneuron pair; MN, motor neuron pair; pr-AMG RN, photoreceptor ascending motor ganglion neuron relay neurons; pr-cor RN, photoreceptor coronet relay neuron; prRN, photoreceptor relay neuron. Reproduced with permission from Ryan and Meinertzhagen (2019).

Finally, Kerrianne Ryan completed this exemplary body of work by elucidating, mainly through serial-section electron microscopy, the entire network of synaptic connections (connectome) that exist between the 177 neurons that, on average, compose the CNS of the *Ciona* larva (Ryan et al., 2016). This study also provided the first evidence of left–right asymmetry in the brain of this simple chordate larva, whereby neurons and connections are different on the sides of the larva (Ryan et al., 2016, 2017). This remarkable work was extended to the *Ciona* peripheral nervous system (PNS), and the synaptic connections between the papillary neurons of the adhesive organ, as well as those between the epidermal neurons, were elucidated in detail (Ryan et al., 2018). In turn, the identification of the PNS connectome shed light on the connections responsible for the swimming movements (Ryan et al., 2018). Together, these studies led to the conclusion that the larval CNS contains ~177–180 neurons of about 50 distinguishable types, each of which forms ~49 connections with other neurons, thus giving rise to an unexpectedly complex network of interactions (Ryan & Meinertzhagen, 2019; Figure 11).

Of note, the *Ciona* CNS was also studied by Kaoru S. Imai during her residency as a visiting scientist in Mike Levine’s lab, which she describes in her own article in this Special Issue. Kaoru analyzed the expression patterns of evolutionarily conserved markers of vertebrate CNS compartments and set forth the hypothesis that a primordial version of the midbrain-hindbrain boundary might be present in the ascidian larval CNS (Imai et al., 2009).

On the other hand, the molecular mechanisms responsible for patterning the *Ciona* PNS are the subject of studies by Agnès Roure, a researcher affiliated with the Observatoire Océanologique of Banyuls-sur-Mer (France). By studying the expression of different genes encoding transcription factors, Agnès determined that two of them, Msxb and Nkx-C, are able to induce the formation of epidermal sensory neurons, and that the number of neurons that form in the larva is dependent upon the Delta/Notch pathway (Roure and Darras, 2016).

Work on the postmetamorphic nervous system of juvenile and adult ascidians was pioneered in the late 1970s by Danielle Georges, a researcher affiliated with the Université Scientifique et Médicale of Grenoble (France) with her research on the *Ciona* nervous ganglion, which is part of the neural complex of the adult (Georges, 1977). She also studied the effects of short treatments with monosodium glutamate on the amount and activity of the neuropeptides present in the ganglion, which she measured indirectly by monitoring the changes in immunoreactivity of the neurons in the ganglion (Georges, 1988). She found that the damage caused by the treatment with monosodium glutamate was similar to the damage caused by the removal of the ovary, and from these observations formulated the hypothesis that through the nervous ganglion, the ovary influences the activity of the neural gland (Georges, 1988). Using immunofluorescence, Danielle found the first evidence of the production of adrenocorticotropin-like substance(s) in the neural complex of adult *Ciona* ascidians (Georges & Dubois, 1979). Over two decades later, her findings were confirmed by studies carried out in another solitary ascidian, *Halocynthia roretzi* (Kawahara et al., 2002).

### 2.5 | *Botryllus schlosseri* research at the Stanford University Hopkins Marine Station

Dr. Virginia (Ginny) Scofield (1948–2017) obtained her PhD in Immunology and Microbiology in 1978 from the University of Texas at Austin (USA). In 1977, she started a postdoctoral position in Prof. Irv Weissman’s laboratory at Stanford University. Dr. Scofield was inspired by the work of Japanese ascidiologists Oka and Watanabe (e.g., Oka & Watanabe, 1957) on the fusion and rejection reactions between colonies of the ascidian *Botryllus primigenus*. Dr. Scofield suggested that Dr. Weissman open a laboratory at the Hopkins Marine Station in Pacific Grove, CA (USA) to study the evolution and genetics of this allorecognition system in the local species *B*. *schlosseri*. Five years later, Dr. Scofield’s work resulted in a paper published in *Nature* describing the highly polymorphic single locus that controls allorecognition in *B*. *schlosseri* (Scofield et al., 1982). Dr. Scofield moved to her own laboratory in 1983, and spent her career studying immunology and cancer biology (Dr. Virginia Scofield). The *B*. *schlosseri* work started by Dr. Scofield in the Weissman Laboratory continues today, with the work of Dr. Ayelet Voskoboynik (featured in this Special Issue) and colleagues. None of the work in the *Botryllus* laboratory at Hopkins Marine Station over the past 40 years would have been possible without two very talented laboratory managers/research technicians who were hired in 1984–1985, Kathy Ishizuka and Karla Palmeri. These two researchers have been responsible for developing the mariculture facilities, writing protocols to care for the animals, conducting genetic crosses, and many other molecular biology techniques specific to *Botryllus*. Ms. Ishizuka and Ms. Palmeri have also mentored countless graduate students, postdoctoral research associates and visiting students over the years. They have contributed substantially to numerous publications on a variety of *B*. *schlosseri*-related topics, including genome sequencing (e.g., Voskoboynik et al., 2013), stem cell biology (e.g., Voskoboynik et al., 2008), and developmental biology (e.g., Kowarsky et al., 2021).

### 2.6 | Françoise Gaill: Active 1972 to present

A prominent oceanographer widely known for her decades-long research in deep-sea ecology and her current advocacy for conservation and sustainability in world oceans (news.cnrs.fr/articles/francoise-gaill-the-voice-of-the-oceans), a chief focus of Dr. Gaill’s early career, from the 1970s to early 1980s, was on the biology of ascidians. Much of this early work addressed problems of ascidian physiology, a nearly neglected topic in recent ascidian research. Dr. Gaill’s early ascidian research focused particularly on the physiology of digestion, especially on development and physiology of the pyloric gland, as well as other aspects of ascidian biochemistry and physiology, such as osmoregulation. Her interests in ascidian physiology and deep-sea biology have converged in several instances, including a 1979 study of the digestive structures of carnivorous deep-sea ascidians (Gaill, 1979).

## 3 | TAXONOMY

A necessary foundation for research on any organism is the ability to identify and describe the organism(s) that one is investigating. Tunicates pose special challenges for this fundamental task, because many tunicate species cannot be distinguished from one another—or even, in some cases, even placed into a genus or family—by relying on external characteristics alone. Adding to this difficulty is the fact that, compared with other major groups of animals, tunicates remain an understudied topic of research in general. As both cause and consequence of this lack of knowledge, documentation of the diversity and distribution of ascidians is incomplete at best (Shenkar & Swalla, 2011); even less is known about ascidians’ oceangoing relatives, the Thaliacea (salps, doliolids, and pyrosomes) and Appendicularia (also known as Larvacea; Holland, 2016). As a symptom of the deficient state of the art, the most comprehensive taxonomic monograph on ascidians of North and South America remains the 1945 treatise by Van Name (1945). Nevertheless, solid taxonomic descriptions of the anatomy, diversity, and distribution of these animals are the essential foundation for resolving the phylogeny of tunicate clades, and for accurate analysis and understanding of the full biological implications of developmental, physiological, or ecological characteristics of particular tunicate species.

Two women, Patricia Kott and Françoise Monniot, have played prominent roles in redressing this deficiency, each with substantive contributions to ascidian taxonomy of the ascidians.

### 3.1 | Patricia Kott Mather (published as Patricia Kott and Patricia Mather), 1925–2012

**FIGURE 12:**
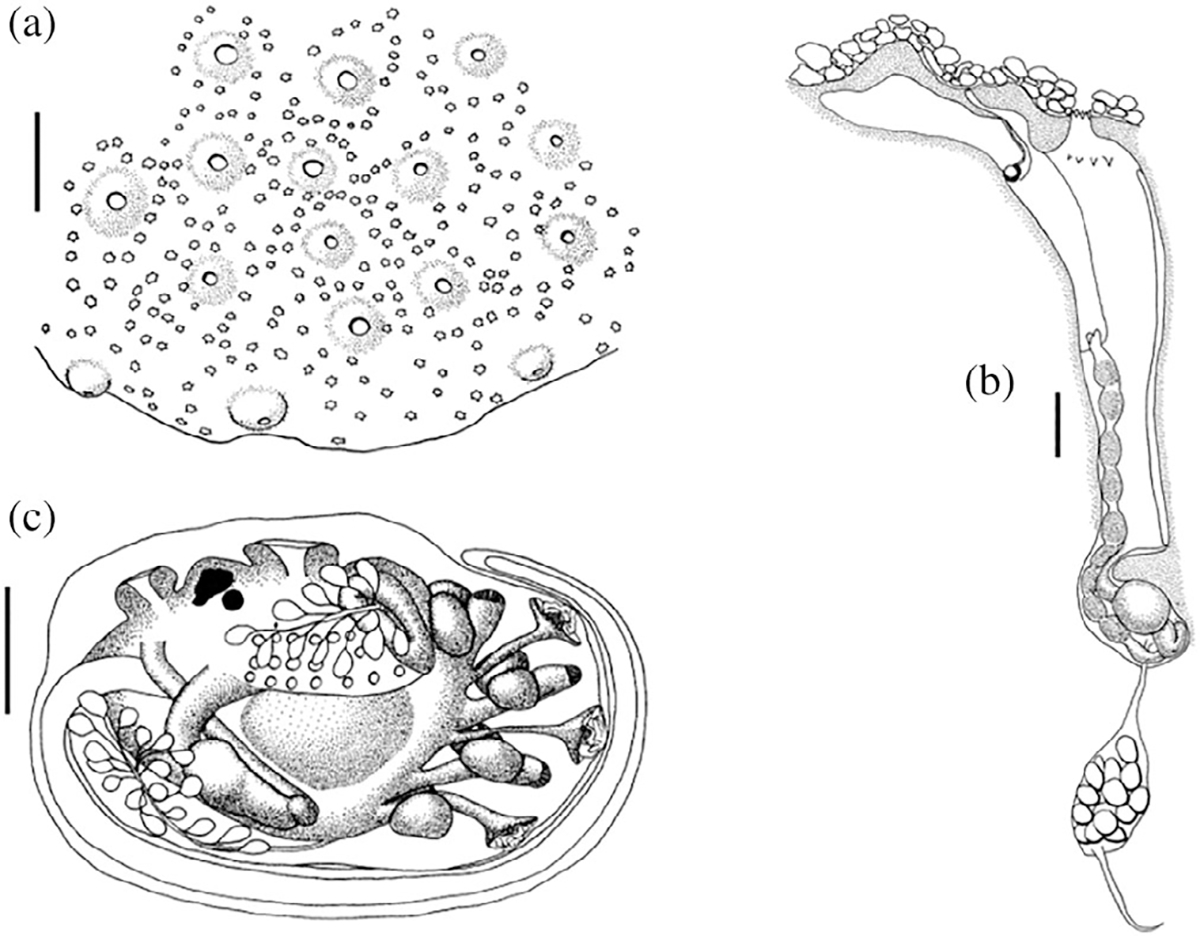
*Polyclinum* spp. drawn by Patricia Kott. (a) colony surface, (b) zooid in tunic, with sand on the surface of the tunic, and (c) larva. From Kott (1992).

Born and raised in Western Australia, Dr. Kott became interested in marine invertebrates as an undergraduate student, and developed expertise in plankton biology during a stint with CSIRO (The Commonwealth Scientific and Industrial Research Organization). During a 2-year period of study and research in England, she started her investigations on ascidian biology; after her return to Australia in 1951, she began to work on ascidian taxonomy, which remained her research focus for the rest of her long career. She published the first 126-page section of her four-part monograph, “The Ascidians on Australia,” a year later (Kott, 1952), followed by the next three parts in 1957, 1962, and 1963 (Kott, 1957, 1962, 1963), her productivity barely slowed by her 1955 marriage to geneticist W. B. Mather and the birth of three children over the next 8 years. She received a PhD from the University of Queensland in 1962 and a DSc from the University of Western Australia in 1970, after she had published two more major monographs on Antarctic ascidians (Kott, 1969; Kott, 1971). From 1985 to 2001, she produced an additional four-part monograph on Australian ascidians (Kott, 1985, 1990, 1992, 2001; Figure 12), an achievement in which she was said to take special pride (Davie, 2012). She also wrote a regular stream of shorter articles, which she continued to publish until a year before her death at age 87. Her husband having predeceased her in 1987, she left behind her children, grandchildren, successful conservation efforts on behalf of the Great Barrier Reef, and the Queensland Museum ascidian collection, which she developed from “a modest holding into the most significant collection of Australian and Indo-West Pacific species in the world” (Davie, 2012). When asked about her proudest achievements, Dr. Kott offered two: her description of 500 Australian ascidian species (more than 70% of the total number of Australian ascidians) and her three sons.

Far from the caricature of taxonomists (and taxonomy) as “dry,” colleagues characterized Dr. Kott with vivid descriptors such as “charming, passionate, persistent, indomitable, focused, demanding, devoted,” (Davie, 2012), “a little bit intimidating” (Hutchings et al., 2023) and a “hard taskmaster” (Davie, 2012) yet “generous,” and “an excellent cook and entertainer” (Hutchings et al., 2023). Her life has been celebrated in a comprehensive biographical article by Davie (2012).

### 3.2 | Françoise Monniot: Active 1961 to present

**FIGURE 13:**
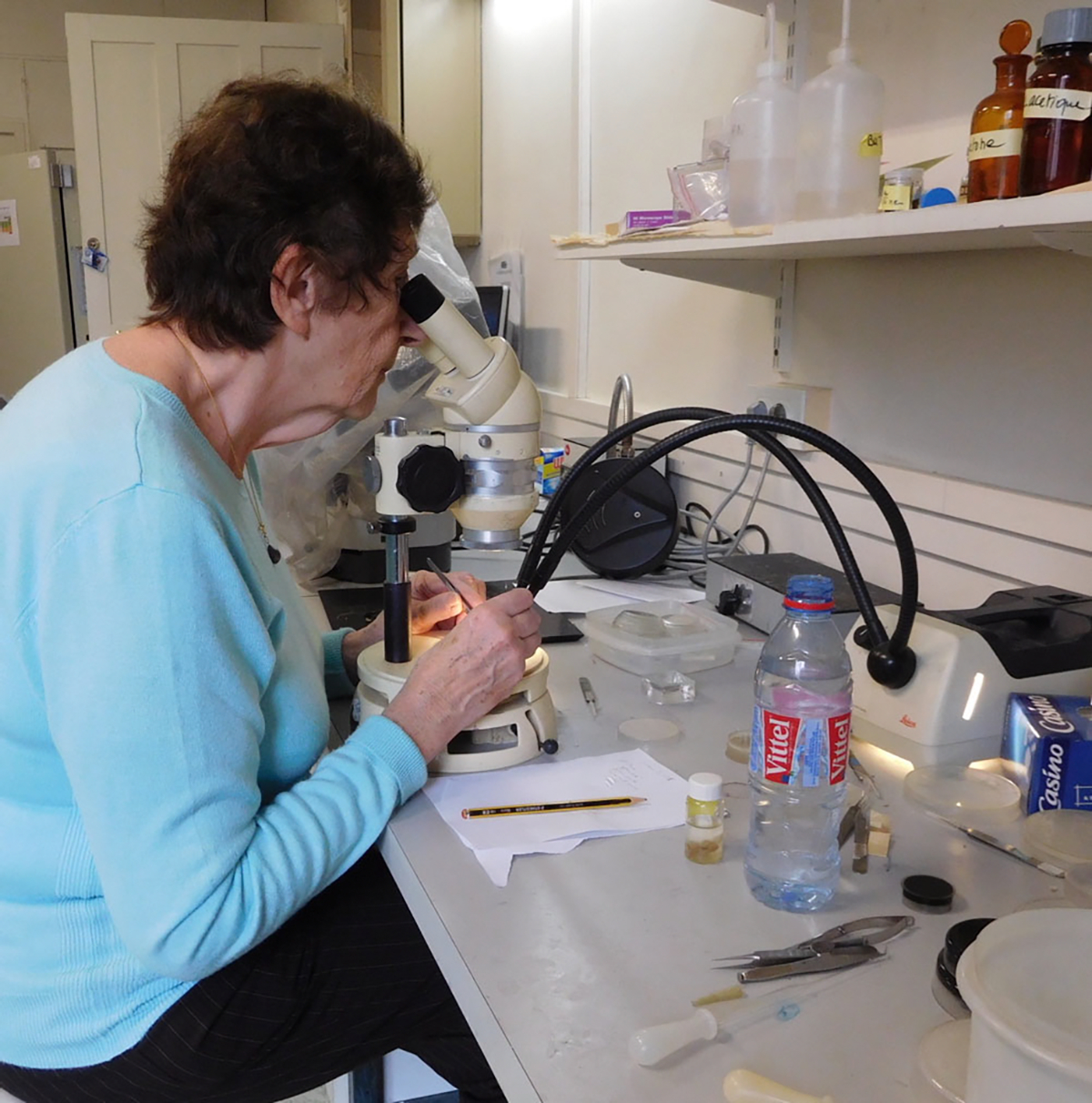
Françoise at work in her laboratory in 2016.

Early in her ascidian taxonomy studies Françoise Monniot concentrated on the almost completely unknown interstitial ascidians, and in 1965 published her PhD thesis: “Ascidies interstitielles des côtes d’Europe” (Monniot, 1965). For much of her career Françoise Monniot worked with her husband Claude Monniot, in a productive, influential, and internationally recognized partnership that has added importantly to our understanding of the taxonomy and distribution of ascidians in world oceans, especially in the tropics and the deep sea (Figure 13).

**FIGURE 14:**
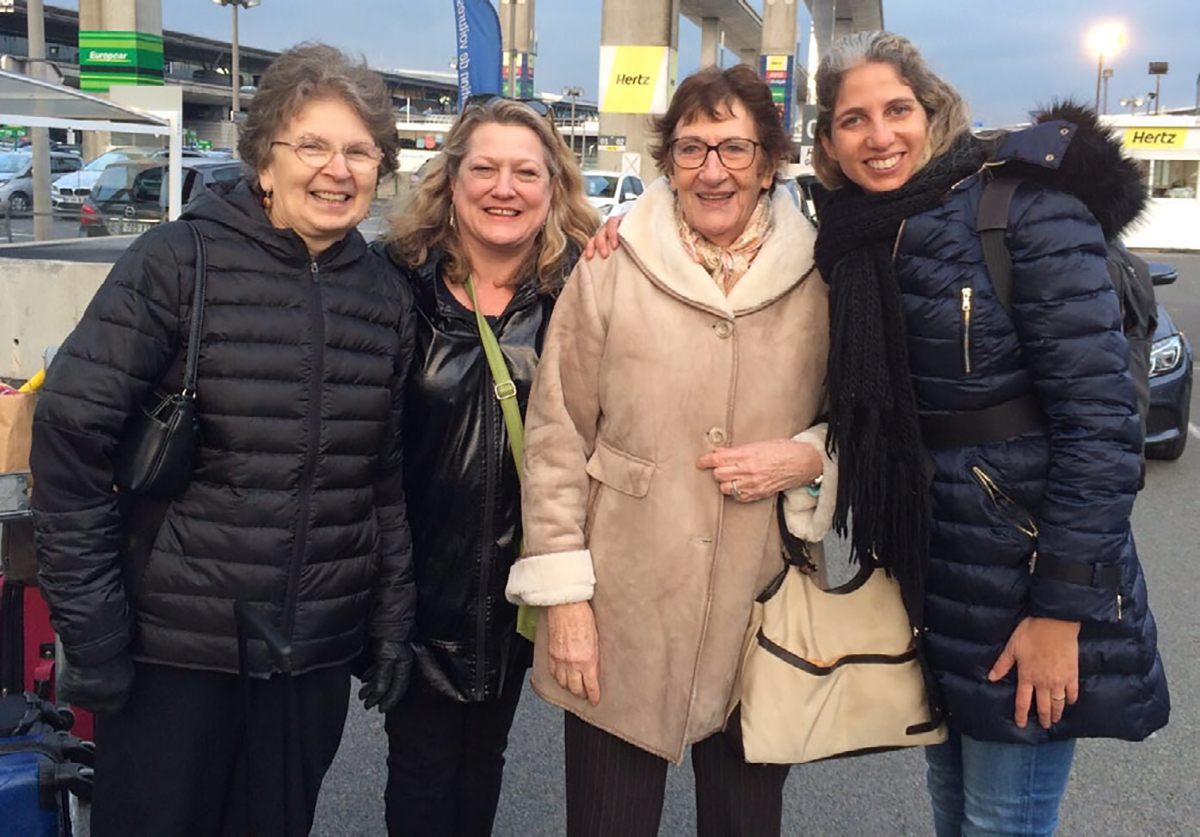
Gretchen Lambert, Billie Swalla, Françoise Monniot, and Noa Shenkar at the WoRMS (World Register of Marine Species) Ascidiacea database editors meeting in Belgium, 2016.

The Monniots’ 1991 book, *Coral Reef Ascidians of New Caledonia* (Monniot et al., 1991), transcends its particular biogeographic focus to serve also as a comprehensive and integrative account of general ascidian biology. Since her husband’s death in 2008 (reported in “Ascidian News”), Françoise Monniot has continued to expand our knowledge of ascidians with numerous papers on the distribution and taxonomy of ascidians from several continents, including Europe, Africa, the Caribbean, west Pacific, the Antarctic and Southern Oceans, and several recent papers on abyssal ascidians from the tropical West Pacific (Monniot, 2021; Monniot, 2022; Monniot & Lopez-Legentil, 2017). With hundreds of highly cited papers as coauthor, first author, or sole author, her impact on the field of ascidian biology was succinctly summarized in an account of a meeting of the ascidiologist taxonomic editors and contributors to the WoRMS (World Register of Marine Species) database (Figure 14), where she was described with pinpoint accuracy as “the reigning world expert on ascidian taxonomy” (Lambert & Swalla, 2016, April http://depts.washington.edu/fhl/tidebites/Vol32/index.html).

**FIGURE 15:**
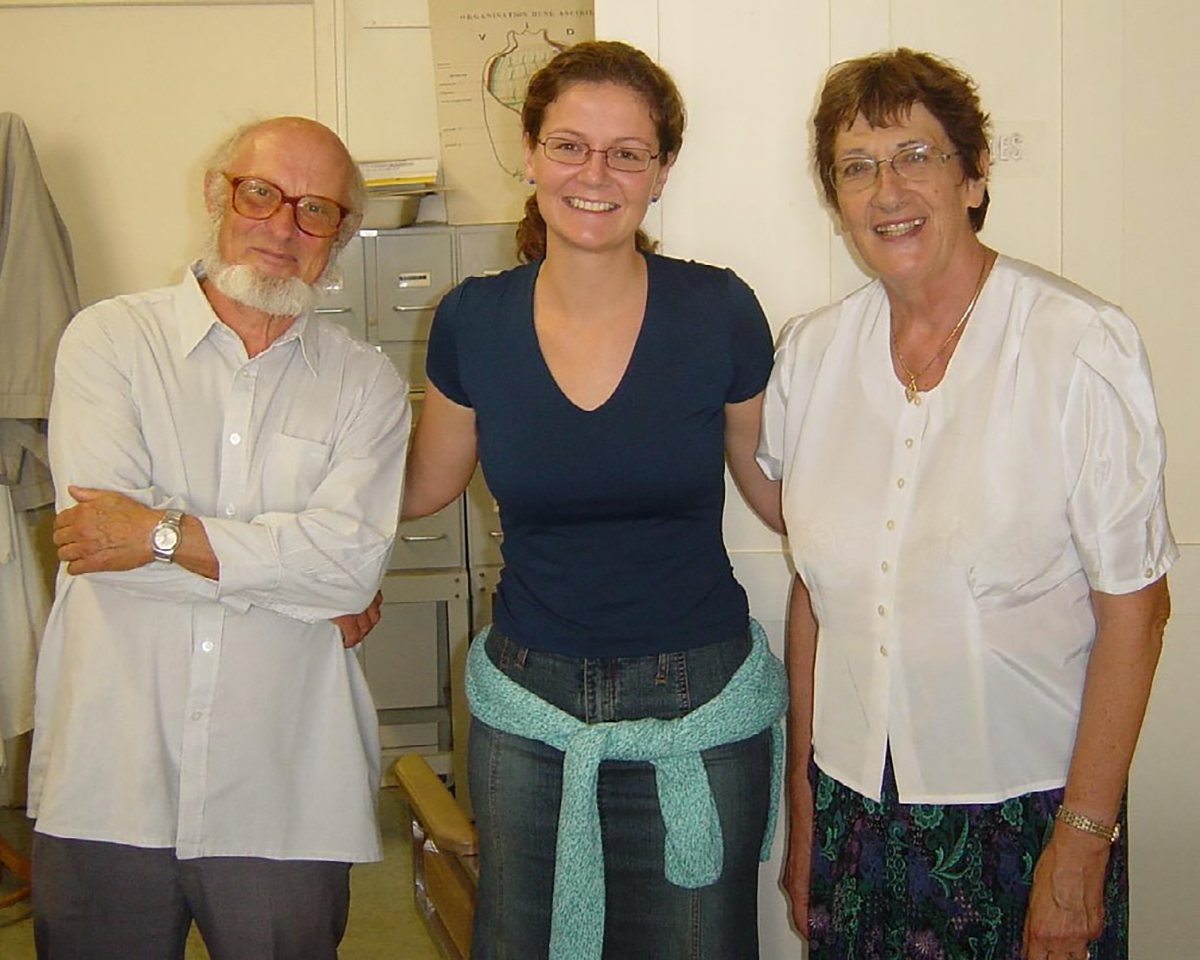
Susanna López-Legentil (center) with Claude and Françoise Monniot in their office at the Muséum National d’Histoire Naturelle in Paris.

Rosana Rocha was a trainee in Françoise and Claude’s laboratory in the 1980s. She remembers that they were very fond of a dog that would come into the laboratory with them—not a very common sight at that time. As part of her research for the first molecular tunicate phylogeny (Hadfield et al., 1995), Billie Swalla (who is featured in her own article in this Special Issue) and her collaborators were in Roscoff, France, collecting ascidians. Billie could tell that there were several species of *Molgula* among the many ascidians they had collected, but *Molgula manhattensis* and *Molgula socialis* were so similar that she could not tell the difference between them. Hence, they packed up several specimens and shipped them to Françoise for identification. She sent them back, beautifully dissected and stained, accompanied by precise, authoritative species identifications based on sperm duct morphology. Billie remembers Dr. Monniot’s generous service as a lovely way for an expert to assist a postdoc who was just learning taxonomy at the time. While still a graduate student, Susanna López-Legentil (who is featured in her own article in this Special Issue) traveled to the Muséum National d’Histoire Naturelle in Paris to meet Françoise and Claude. Susanna fondly remembers both of them being very kind and supportive of her research. (Figure 15). In addition to her substantive body of work and her mentorship of many younger scientists, Françoise’s other biological contributions include a daughter and two granddaughters.

### 3.3 | Françoise Lafargue: Active 1968–1992

**FIGURE 16:**
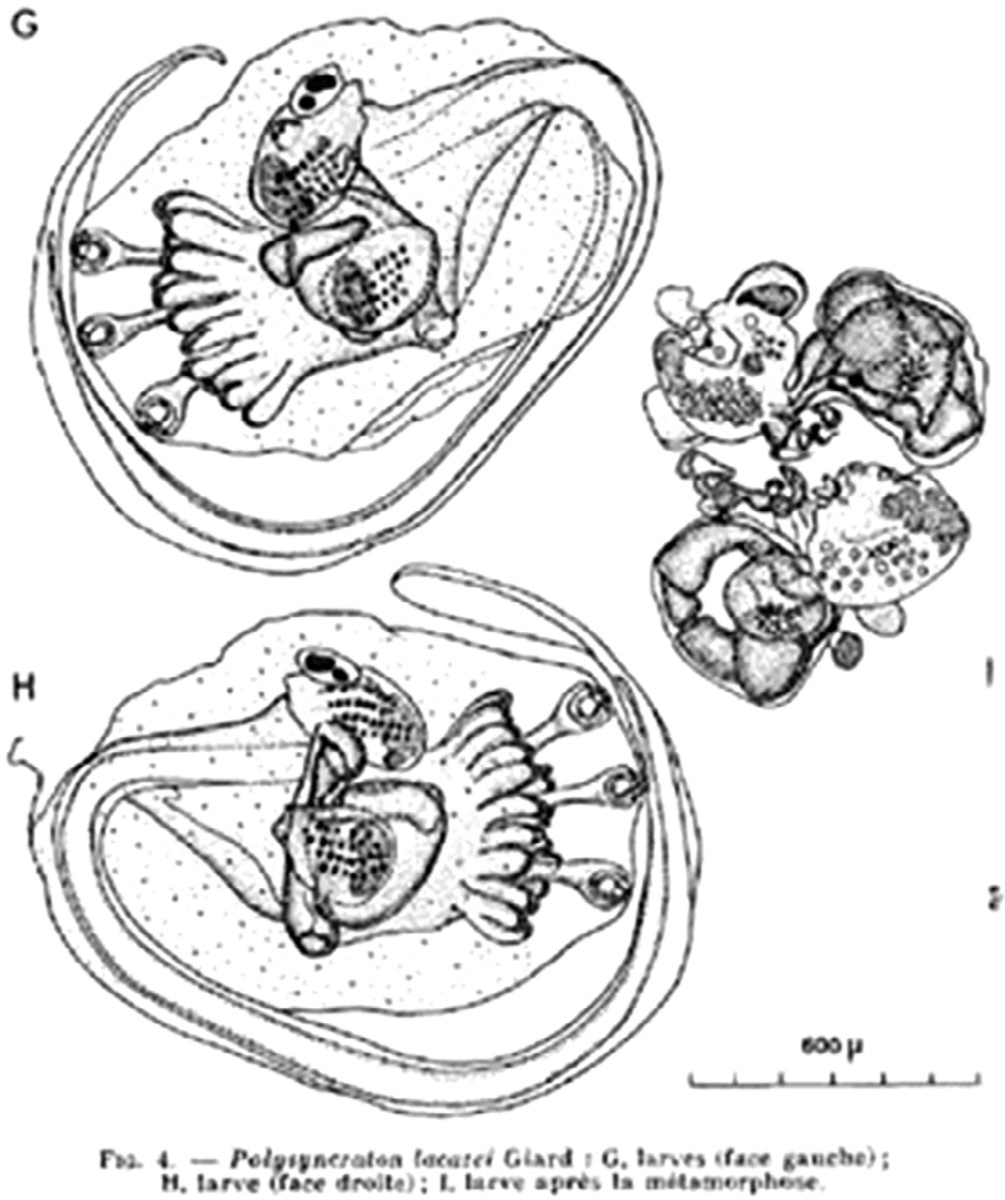
*Polysyncraton lacazei* larva. G (left view), H (right view), and I (during metamorphosis). From Lafargue (1975a).

Françoise Lafargue was a researcher at the Observatoire Océanologique de Banyuls-sur-Mer (France, also known as the Laboratoire Arago) in Banyuls-sur-Mer, France. She primarily published on taxonomy, evolution, and ecology of ascidian species on the French coasts (Castric-Fey et al., 1973, 1978; Copello et al., 1981; Lafargue, 1968, 1970, 1975a, 1975b, Lafargue & Wahl, 1987; Wahl & Lafargue, 1990), the Ionian and Adriatic coasts of Italy (Lafargue & Tursi, 1975), the Mediterranean coast of Spain (Lafargue et al., 1986; Ramos et al., 1991), Helgoland (Lafargue, 1972), the Red Sea (Lafargue, 1974), and Senegal (Lafargue and Wahl 1990). Figure 16 provides an example of her beautiful illustrations, in this case the larva of *Polysyncraton lacazei* (Lafargue, 1975a). She also participated in studies of organic compounds found in ascidians (Aracil et al., 1991; Banaigs et al., 1989; Demattè et al., 1985; Rinehart Jr et al., 1987), the characterization of proteins in ascidian sperm (Chiva et al., 1992), spicule formation in didemnid colonies (Lafargue & Kniprath, 1978), algal symbionts in ascidians (Duclaux et al., 1988), and species boundaries in the genus *Ciona* (Lambert et al., 1990). She described several new species, including *Didemnum drachi* (Lafargue, 1975a, 1975b), *Lissoclinum weigelei* (Lafargue, 1968), and *Polysyncraton bilobatum* (Lafargue, 1968).

Xavier Turon, who worked with Françoise in the 1980s, remembers her as an agreeable, cultured person who was a good conversationalist and was very fond of her pet cat. At work, she was very meticulous (as is necessary to study the Family Didemnidae) and committed (X. Turon, personal communication).

A discussion of female ascidian taxonomists would not be complete without Nadya Sanamyan, a Russian researcher who often publishes with her husband, Karen Sanamyan. The majority of Dr. Sanamyan’s articles focus on sea anemones, but she has also contributed to ascidian, hydroid, mollusk, and sponge taxonomy. Importantly, she focuses on geographical areas that are understudied in ascidian taxonomy, including the Northwest Pacific (Kuril Islands between Russia and Japan, Kamchatka Peninsula in Russia’s Far East, and the Commander Islands off the coast of Kamchatka; e.g., Sanamyan & Sanamyan, 2017), Antarctica and the Southeast Pacific (Schories et al., 2015), and Chile (Sanamyan et al., 2010). She and her husband are among the rare ascidian taxonomists who (along with Françoise Monniot) routinely study the morphology and taxonomy of deep sea ascidians (e.g., Sanamyan & Sanamyan, 2012). Dr. Sanamyan often collects her own samples via SCUBA, and publishes exquisite underwater photographs.

## 4 | UNEXPECTED SYMBIONTS

### 4.1 | Mary Beth Saffo: Active 1978 to present

Tunicates challenge biologists on many fronts. While tunicates might be best known for their relevance to vertebrate evolution, or for the unwanted talents of a few species in boat-fouling or species invasions, ascidians also offer many other important lessons to biologists. Perhaps even more important than their relationship to vertebrates is the fact that—as Emeritus U.C. Santa Cruz professor Todd Newberry (a student of Don Abbott and a specialist on colonial ascidians) often reminded his undergraduate students, including Dr. Saffo—tunicates do not obey the rules. The many surprising, and often downright bizarre, characteristics of tunicates remind us that there is much more to biology than the standard “laws” generated by inbred mice, *Caenorhabditis elegans*, *Drosophila*, and *Escherichia coli*. As difficult animals to study, and in raising head-scratching questions for those biologists who succeed in persuading them to grudgingly reveal some of their secrets, tunicates prod us to think a little harder about how life really works outside the controlled world of laboratory models (Yoon, 1994).

While a graduate student at Stanford University, Dr. Saffo became intrigued by one of the several ascidian organs whose function is poorly understood: a ductless organ in molgulid ascidians, the “renal sac,” whose development, morphology, and kidney stone-like urate/calcium oxalate contents (Saffo & Lowenstam, 1978) did not fit the textbook notions of a normal kidney, especially for marine animals. Like molgulids, most ascidians harbor urate (and at least in some cases, calcium) deposits in blood cells or small closed vesicles, and the biological significance of this physiological habit remains unclear. But the molgulid renal sac is distinct from its counterparts in other ascidians in one big way: its colonization, in nearly 100% of all adult molgulids (Saffo, 1982, 1991), by symbiotic microbes, which Dr. Saffo discovered as a graduate student—or rather rediscovered, as similar fungus-like microbes had been described 100 years earlier by the French biologist Henri de Lacaze-Duthiers and later dubbed “*Nephromyces*”; but the strangeness of the early descriptions had led subsequent biologists to doubt *Nephromyces’* very existence. Saffo et al. (2010) have now shown that *Nephromyces* is an apicomplexan—a distant relative of malaria parasites (Muñoz-Gomez et al., 2019)—a surprising result given the fact that the Apicomplexa have been traditionally considered an exclusively parasitic clade, and *Nephromyces* appears to be a mutualistic symbiont of molgulids; strikingly, however, its closest relative is an apicomplexan parasite of ascidians, but with a variable but widespread distribution among a broad array of ascidian species, including *Ciona* (Saffo et al., 2010; Zimmer, 2010). Work by Saffo, her student Brandon Seah, and collaborators, has revealed more complexities to the *Nephromyce*s-molgulid symbiosis: *Nephromyces* itself harbors intracellular bacterial symbionts belonging to several taxa (Paight et al., 2022; Saffo, 1990; Saffo et al., 2010; Seah, 2011).

The ubiquity of *Nephromyces* (and bacteria) in molgulids suggests that these ascidians have coevolved with their microbial symbionts, with apparently significant consequences for both hosts and symbionts. Both molecular sequencing and morphological characteristics indicate that *Nephromyces* is a biologically unique clade, and the Monniots, Billie Swalla, and others similarly suggest that molgulids are a genomically, developmentally, morphologically, and ecologically distinctive group of ascidians. Has symbiosis led both the animal and microbial partners on novel evolutionary paths? Finding answers will require not only genomics, but also renewed attention to the host organisms, to fully unravel the biological consequences and ecological context of this surprising ascidian-microbial partnership.

## 5 | ECOLOGY

### 5.1 | Alice Alldredge (active 1972–2020) and Mary Silver (active 1971–2012)

If ascidians are puzzling animals, the even stranger biology of their pelagic tunicate relatives invites hypotheses about the possibilities of alien life. But, the contributions of Alice Alldredge (Professor Emerita, U.C. Santa Barbara) and Mary Silver (Professor Emerita, U.C. Santa Cruz) to tunicate biology have established the genuine ecological importance of these surprising animals. Neither Drs. Alldredge or Silver would identify themselves as “tunicateologists,” despite the facts that Dr. Silver focused her PhD thesis on the ecology of salps in the California Current, and that key research accomplishments of both women have involved important discoveries relating to appendicularian biology. Nevertheless, the seminal work of these biological oceanographers on pelagic tunicates has had profound effects on our understanding of the ecology of the oceans, and of pelagic tunicates themselves, especially of appendicularians. The careers of both women are sprinkled with awards and a series of “firsts.” Most notably, they are the first, and thus far, only women to have won the Bigelow Prize, periodically awarded by the Woods Hole Oceanographic Institution (WHOI) for outstanding contributions to oceanography (WHOI, 2023); Profs. Alldredge and Silver were cited jointly for their pioneering work on “marine snow,” whose larger particles consist largely of discarded “houses” of appendicularians (Alldredge, 1976; Silver, 2015; Silver et al., 1978; Trent et al., 1978). In 2002, Prof. Silver was recognized as a women pioneer in oceanography by WHOI’s Mary Sears Award (Silver, 2005); in 2009, Prof. Alldredge won the G. Evelyn Hutchinson award from the American Society of Limnology and Oceanography for excellence in limnology or oceanography research.

Notably, Alldredge, Silver, and colleagues (including two of Mary Silver’s undergraduate research students, Alan Shanks and Jonathan Trent) were able to make their crucial discoveries not because of unusual technical instrumentation or elaborate remote sensors, but instead, simply because they paid attention, collecting their data by direct SCUBA-enabled in situ observation in the open ocean and hand collecting the fragile samples for later analysis in the laboratory. In doing so, they have not only opened up new paths for future research on the fascinating biology of pelagic tunicates: their work has highlighted pelagic tunicates not just as interesting ocean oddities, but instead as important contributors to nutrient flux in ocean ecosystems.

### 5.2 | To be continued…

The gallery of scientific portraits that we have begun to sketch here is far from complete, and farther from final. We are well aware that there are several other women who have poured their time, effort and studies to the cause of shedding light on the ever-surprising biology of these captivating distant relatives of ours. And to these women, and the many contemporary colleagues that we have not been able to honor here, we dedicate this manuscript, which unavoidably, and promisingly, remains “in progress.”
